# A Neurotoxic Glycerophosphocholine Impacts PtdIns-4, 5-Bisphosphate and TORC2 Signaling by Altering Ceramide Biosynthesis in Yeast

**DOI:** 10.1371/journal.pgen.1004010

**Published:** 2014-01-23

**Authors:** Michael A. Kennedy, Kenneth Gable, Karolina Niewola-Staszkowska, Susana Abreu, Anne Johnston, Linda J. Harris, Fulvio Reggiori, Robbie Loewith, Teresa Dunn, Steffany A. L. Bennett, Kristin Baetz

**Affiliations:** 1 Ottawa Institute of Systems Biology, Department of Biochemistry, Microbiology, and Immunology, University of Ottawa, Ottawa, Ontario, Canada; 2 Department of Biochemistry, Uniformed Services University of the Health Sciences, Bethesda, Maryland, United States of America; 3 Department of Molecular Biology and Swiss National Center for Competence in Research Programme Chemical Biology, University of Geneva, Geneva, Switzerland; 4 Department of Cell Biology and Institute of Biomembranes, University Medical Center Utrecht, Utrecht, The Netherlands; 5 Eastern Cereal and Oilseed Research Centre, Agriculture and Agri-Food Canada, Ottawa, Ontario, Canada; Dalhousie University, Canada

## Abstract

Unbiased lipidomic approaches have identified impairments in glycerophosphocholine second messenger metabolism in patients with Alzheimer's disease. Specifically, we have shown that amyloid-β42 signals the intraneuronal accumulation of PC(*O*-16:0/2:0) which is associated with neurotoxicity. Similar to neuronal cells, intracellular accumulation of PC(*O*-16:0/2:0) is also toxic to *Saccharomyces cerevisiae*, making yeast an excellent model to decipher the pathological effects of this lipid. We previously reported that phospholipase D, a phosphatidylinositol-4,5-bisphosphate (PtdIns(4,5)P_2_)-binding protein, was relocalized in response to PC(*O*-16:0/2:0), suggesting that this neurotoxic lipid may remodel lipid signaling networks. Here we show that PC(*O*-16:0/2:0) regulates the distribution of the PtdIns(4)P 5-kinase Mss4 and its product PtdIns(4,5)P_2_ leading to the formation of invaginations at the plasma membrane (PM). We further demonstrate that the effects of PC(*O*-16:0/2:0) on the distribution of PM PtdIns(4,5)P_2_ pools are in part mediated by changes in the biosynthesis of long chain bases (LCBs) and ceramides. A combination of genetic, biochemical and cell imaging approaches revealed that PC(*O*-16:0/2:0) is also a potent inhibitor of signaling through the Target of rampamycin complex 2 (TORC2). Together, these data provide mechanistic insight into how specific disruptions in phosphocholine second messenger metabolism associated with Alzheimer's disease may trigger larger network-wide disruptions in ceramide and phosphoinositide second messenger biosynthesis and signaling which have been previously implicated in disease progression.

## Author Summary

Accelerated cognitive decline in Alzheimer's patients is associated with distinct changes in the abundance of choline-containing lipids belonging to the platelet activating factor family. In particular, PC(*O*-16:0/2:0) or C16:0 platelet activating factor (PAF), is specifically elevated in brains of Alzheimer's patients. Since elevated intraneuronal levels of PC(*O*-16:0/2:0) are thought to contribute to the loss of neuronal cells it is imperative to identify the underlying mechanisms contributing to the toxic effects of PC(*O*-16:0/2:0). In this study, we have determined that elevated levels of PC(*O*-16:0/2:0) has negative effects upon the distribution of phosphoinositides at the plasma membrane leading to a potent inhibition of target of rapamycin (TOR) signaling. We further show that the changes in phosphoinositide distribution are due to changes in ceramide metabolism. In conclusion, our study suggests that the toxicity associated with aberrant metabolism of glycerophosphocholine lipids species is likely due to the remodeling of phosphoinositide and ceramide metabolism and that therapeutic strategies which target these disruptions may be effective in ameliorating Alzheimer's Disease pathology.

## Introduction

Remodeling of lipid species is required for maintaining normal cellular function and disruptions in lipid homeostasis are believed to contribute to aberrant cellular processes and toxicity associated with specific diseases [1]. Although significant advances have been made in characterizing the changes in lipid composition that occur in pathological conditions, it has proven difficult to connect these changes with relevant signaling networks that regulate cellular growth and viability.

This is especially true for Alzheimer's disease (AD) for which there is increasing evidence that lipid dyshomeostatsis is playing a central role in the disease progression [2], [3]. Recent lipidomic studies on both post mortem brain tissue and AD mouse models have not only detected dramatic changes in lipid species of most of the major lipid subclasses including ceramides, cholesterols, sphingolipids, phosphatidic acids and glycerophospholipids, but have also reported the presence of distinct changes between regions of the brain [4]. Although these dramatic alterations in lipid homeostasis correlate with the disease, it is imperative to identify the specific subspecies that are critical in contributing to the AD pathology by identifying their impact on signaling networks, which contribute to cellular toxicity.

One lipid metabolite with neurotoxic properties that is of particular interest in AD is 1-*O*-hexadecyl-2-acetyl-sn-glycerophosphocholine or PC(*O*-16:0/2:0), also known as C16:0 Platelet Activating Factor (PAF). We have shown that amyloid-β42 signals the intraneuronal accumulation of PC(*O*-16:0/2:0) in AD and that this lipid second messenger, in turn, signals tau-hyperphosphorylation and induces caspase-dependent cell death independently of the G-protein coupled PAF receptor (PAFR) [5]–[7]. However, the underlying signaling pathways mediating the receptor-independent toxicity of PC(*O*-16:0/2:0) remain enigmatic.

The budding yeast *Saccharomyces cerevisiae* has been a valuable tool for identifying basic elements of lipid signaling networks associated with diseases as many of the fundamental processes of lipid metabolism and signaling are remarkably well conserved with mammalian cells [8]. Previously we employed a chemical genomic screen to identify signaling networks involved in regulating the receptor independent toxicity of PC(*O*-16:0/2:0). Using this approach we identified a conserved role for phospholipase D (PLD) (*S. cerevisiae* Spo14) in buffering against the toxicity of PC(*O*-16:0/2:0) in both yeast and cultured neuronal cells [9]. We also reported relocalization of GFP-tagged Spo14 to distinct foci juxtaposed to the PM upon PC(*O*-16:0/2:0) treatment. Since PLD activation and localization depends upon the binding to PtdIns(4,5)P_2_ [10]–[12], our findings suggested that the toxic accumulation of PC(*O*-16:0/2:0) may elicit effects upon signaling networks that regulate the PM distribution of PtdIns(4,5)P_2_.

Here we provide more precise mechanistic insights by showing that PC(*O*-16:0/2:0) promotes the redistribution of the sole yeast PtdIns(4)P-5 kinase, Mss4, which gives rise to the formation of large invaginations of the PM that we have called PtdIns(4,5)P_2-_enriched structures (PES). We also show that PC(*O*-16:0/2:0) remodeling of the PtdIns(4,5)P_2_ PM pool is associated with the potent inhibition of Tor2 signaling. Consistent with these findings we observed that the effects of PC(*O*-16:0/2:0) upon Mss4 distribution and PES formation depend on the accumulation of LCBs and ceramides. Together these findings identify a novel signaling network wherein toxic levels of PC(*O*-16:0/2:0) modulate LCBs and ceramide metabolism, which in turn promotes the redistribution of PM PtdIns(4,5)P_2_ and the inhibition of Tor2 signaling. Our work provides further information into how the toxic accumulation of PC(*O*-16:0/2:0), as observed in AD patients [6], may impact other lipid signaling networks (i.e., ceramide, PtdIns(4,5)P_2_) which have previously been implicated in the progression of this disease [13]–[19].

## Results

### PC(*O*-16:0/2:0) treatment remodels PM PtdIns(4,5)P_2_ distribution

**Figure 1:**
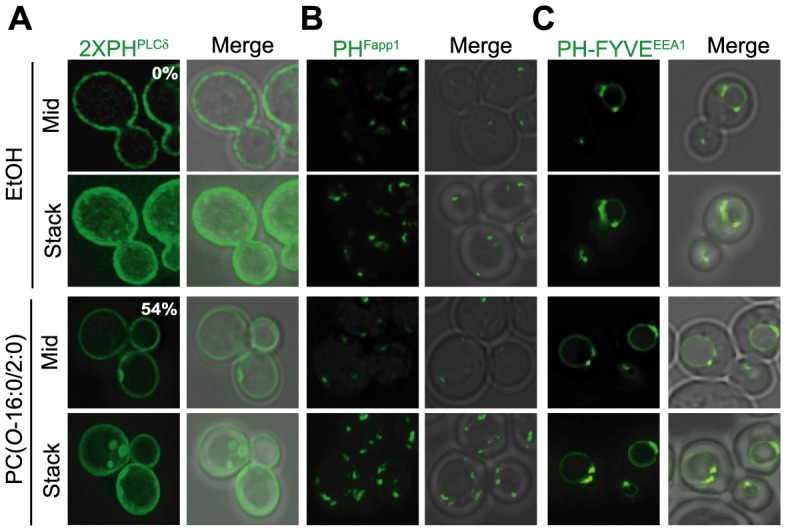
PtdIns(4,5)P_2_ is redistributed in response to PC(*O*-16:0/2:0). Wild type (WT) cells (YPH500) expressing (**A**) GFP-2×PH^PLCδ^ (PtdIns(4,5)P_2_) (**B**) GFP-PH^Fapp^ (PtdIns(4)P) or (**C**) GFP-FYVE^EEA1^ (PtdIns(3)P) were treated with either vehicle (EtOH) or PC(*O*-16:0/2:0) (20 µM, 15 min) and localization of the GFP probe quantified. The percentage of cells displaying a redistribution of the fluorescent reporter is reported in the inset of the figure.

We had previously shown that PC(*O*-16:0/2:0) exposure led to the redistribution of the yeast PLD Spo14 at the PM into discrete foci [9]. As PLD activity is required to buffer the toxic effects of this lipid in both budding yeast and murine N2A neuroblastoma cells [9], we sought to discern the mechanism underlying the changes in PLD distribution. Since the localization of this enzyme to the PM is dependent upon interactions with PtdIns(4,5)P_2_, we examined the effects of PC(*O*-16:0/2:0) on the distribution of this lipid using a fluorescent probe for PtdIns(4,5)P_2_, GFP-2×PH^PLCδ^ (Fig. 1A) [12], [20]–[22]. Similarly to Spo14, growth in the presence of PC(*O*-16:0/2:0) resulted in the relocalization of the GFP-tagged reporter construct to distinct membrane associated structures at the PM which we have termed PtdIns(4,5)P_2_ enriched structures (PES) (Fig. 1A). The appearance of the PES was maximal after 15 min of treatment with PC(*O*-16:0/2:0) and persisted for up to 90 min (Figure S1). This result was specific for PC(*O*-16:0/2:0) as all other related lipids, chemicals and conditions examined did not result in PES formation (Table S1). Furthermore, the distribution of GFP-tagged probes with specificity for additional intracellular phosphoinositides, PtdIns4P (PH^Fapp1^) and PtdIns3P (PH-FYVE^EEA1^), were unaltered by PC(*O*-16:0/2:0) treatment suggesting a specific effect of this lipid on PM PtdIns(4,5)P_2_ (Fig. 1B and C) [20], [23].

### PC(*O*-16:0/2:0) disrupts PM MSS4 distribution

**Figure 2:**
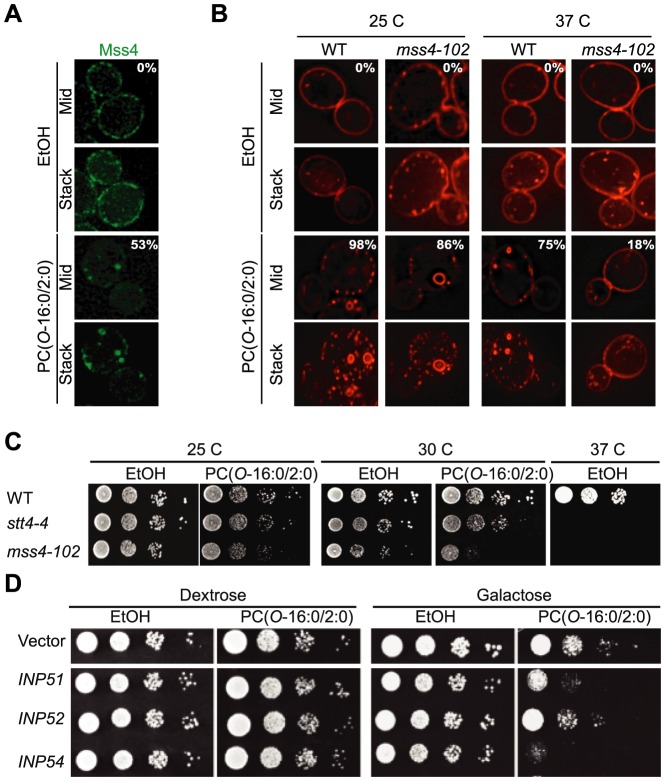
PC(*O*-16:0/2:0)-induced changes in PtdIns(4,5)P_2_ metabolism. (**A**) **Mss4 is relocalized upon PC(*O*-16:0/2:0) treatment.** Mss4-GFP expressing cells (YKB2955) were treated with vehicle (EtOH) or PC(*O*-16:0/2:0) (20 µM, 15 min) and localization examined. Percentage of cells with relocalized Mss4-GFP are indicated by the figure inset. (**B**) **Mss4 is required for PES formation.** Wild type (SEY6210) and *mss4-102* (AAY202) strains were grown at the indicated temperatures for one hour. Cells were subsequently treated with either vehicle (EtOH) or PC(*O*-16:0/2:0) as previously done (20 µM, 15 min). Following treatment cells were collected into ice cold growth media and labeled with FM4-64 in ice cold growth media to visualize the PM. The percentage of cells with PES type structures for each condition are indicated by the figure inset. (**C**) ***MSS4*** **and** ***STT4*** **are required for buffering against PC(*O*-16:0/2:0) toxicity.** The sensitivity of wild strains (SEY6210) or strains expressing a temperature sensitive alleles of either *STT4* (*stt4-4*, AAY102) or *MSS4* (*mss4-102*, AAY202) to PC(*O*-16:0/2:0) was examined by growth on plates containing vehicle (EtOH) or PC(*O*-16:0/2:0) (3 µg/ml or 5.7 µM) for 2 days at permissive (25 C) and semi-permissive (33 C) temperatures. (**D**) **Overexpression of phosphatidylinositol phosphatases increase sensitivity to PC(*O*-16:0/2:0).** The effect of phosphatidylinositol phosphatases upon PC(*O*-16:0/2:0) sensitivity was examined by spotting 10-fold serial dilutions of wild type strain (BY4741) harboring plasmid borne, GAL-inducible *INP51*, *INP52* and *INP54* on plates containing vehicle (EtOH) or PC(*O*-16:0/2:0) (3 µg/ml) with either dextrose or galactose as the carbon source.

The abundance of PtdIns(4,5)P_2_ depends upon the opposing actions of Mss4 and multiple PtdIns(4,5)P_2_ phosphatases including Inp51, Inp52 and Inp54 (reviewed in [24]). Similar to our previous findings with GFP-tagged Spo14 and GFP-2×PH^PLCδ^, PC(*O*-16:0/2:0) treatment resulted in the relocalization of Mss4-GFP to distinct foci within the cell (Fig. 2A). This result suggested that PC(*O*-16:0/2:0)-induced PES formation requires Mss4 activity. To investigate this possibility, we assessed PC(*O*-16:0/2:0)-induced PES formation in wild type cells and those carrying a thermosensitive allele of *MSS4* (*mss4-102*) [20]. The reduced levels of PtdIns(4,5)P_2_ in *mss4-102* cells precluded the use of GFP-2×PH^PLCδ^ [20]–[22]. Therefore, changes in the PM structure were visualized using the lipophillic probe FM4-64, which co-localizes with GFP-2×PH^PLCδ^ following PC(*O*-16:0/2:0) treatment in wild type cells (Fig. S2). As expected, both wild type and *mss4-102* cells grown at the permissive temperature (25 C) exhibit similar FM4-64 labeling that was restricted to the PM and early endosomes in untreated cells (Fig. 2B). Following treatment with PC(*O*-16:0/2:0), structures similar to the PES were observed to form in both strains (Fig. 2B). Growth at the restrictive temperature did not impact PES formation in wild type cells as the formation of these structures was similar to previous results with maximal PES formation evident at 15 min and persisting for at least 60 min (Fig. 2B and Fig. S2E). However, PES formation was reduced in *mss4-102* cells at all examined time points (Fig. 2B and Fig. S2E) suggesting that Mss4 activity is involved in PES formation. To assess the significance of Mss4-dependent PtdIns(4,5)P_2_ synthesis in buffering against PC(*O*-16:0/2:0) toxicity, we examined the growth of strains possessing temperature sensitive alleles of *MSS4* (i.e. *mss4-102*) and the PtdIns 4-kinase *STT4* (i.e. *stt4-4*) [20], [25]. Both mutant strains displayed increased sensitivity to PC(*O*-16:0/2:0) compared to the isogenic wild type control whereas overexpressing Mss4 reduced the growth inhibitory effects of PC(*O*-16:0/2:0) in an otherwise wild type strain (Fig. 2C and Fig. S2F). Furthermore, growth was also impacted by reducing or increasing the cellular PtdIns(4,5)P_2_ levels through overexpressing or deleting phosphoinositide phosphatases respectively (Fig. 2D and Fig. S2G–H). In particular, overexpression of Inp51 and Inp54 resulted in reduced growth whereas deletion of Inp51 alone improved growth in the presence of PC(*O*-16:0/2:0) (Fig. 2D and Fig. S2G) [24]. Together these results indicate that cellular PtdIns(4,5)P_2_ and PES formation are important for buffering against the toxic effects of PC(*O*-16:0/2:0).

### The PES are PM invaginations that form independently of the actin cytoskeleton

**Figure 3:**
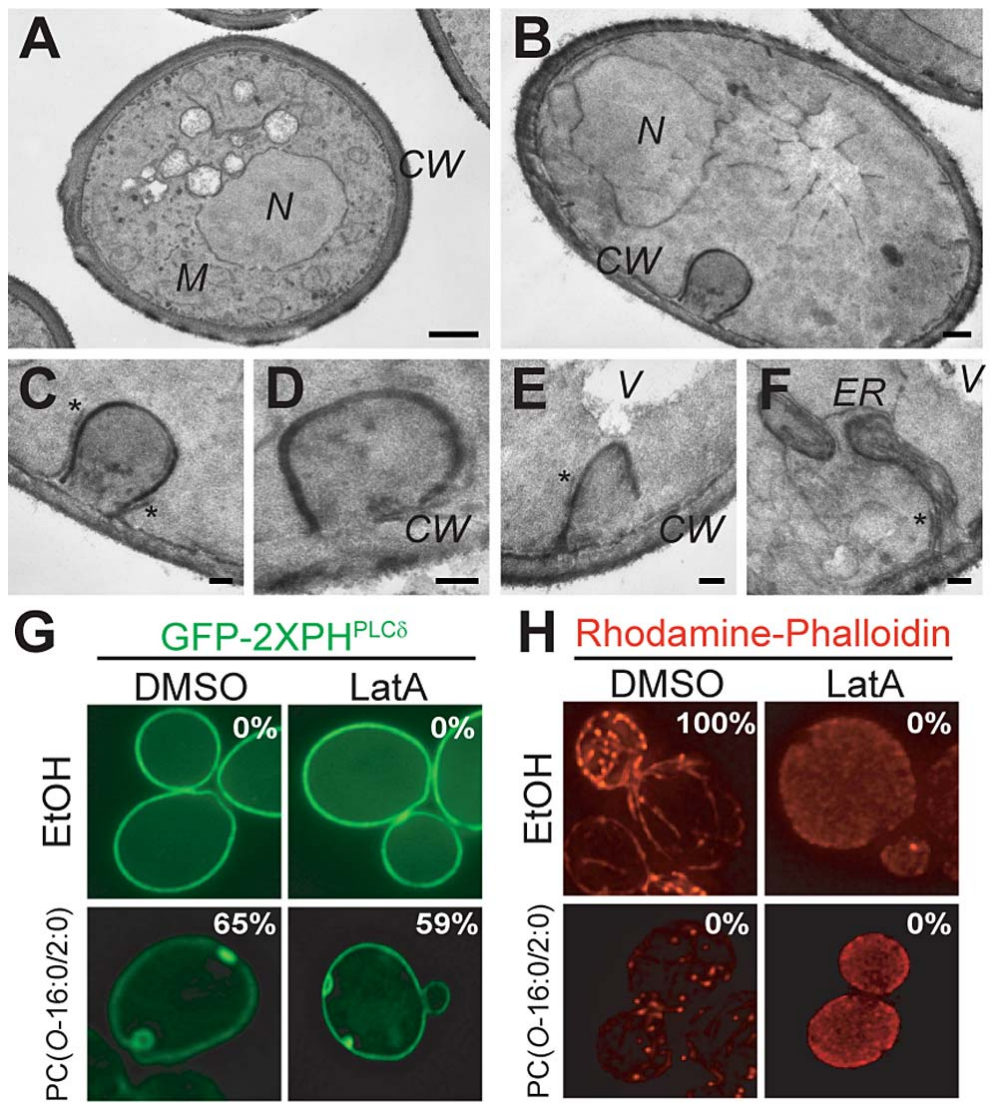
Characterization of PM changes in PC(*O*-16:0/2:0)-treated cells. **Large PM invaginations are present in PC(** ***O*** **-16:0/2:0)-treated cells.** Wild type cells (BY4742) exposed (**B, C, D, E and F**) or not (**A**) to PC(*O*-16:0/2:0) for 15 min were processed for EM as previously described [55]. Panel (**C**) is an inset of panel (**B**). Panels (**D**), (**E**) **and** (**F**) show magnifications of the large PM invaginations induced by PC(*O*-16:0/2:0), which very likely represent the PES. The asterisks indicate the peripheral ER that is associated with the PM. CW, cell wall; ER, endoplasmic reticulum; M, mitochondrion; V, vacuole. Bars in panels (**A**) and (**B**), 500 µm; bars in panels (**C**), (**D**), (**E**) **and** (**F**), 100 µm. **PES formation still occurs in the presence of depolymerised actin.** (**G**) Wild type cells (YPH500) expressing GFP-2×PH^PLCδ^ were treated with Latrunculin A (5 µM, 30 min) to induce depolymerization of the actin cytoskeleton prior to treatment with PC(*O*-16:0/2:0) (20 µM, 15 min) and imaged live. The percentage of cells displaying a redistribution of the fluorescent reporter is reported in the inset of the figure. (**H**) An aliquot of cells was also fixed following treatment for imaging of the actin cytoskeleton by staining with Rhodamine-conjugated phalloidin. The percentage of small budded cells displaying a polarized actin cytoskeleton is reported in the inset of the figure.

We next sought to investigate the cellular processes involved in PES formation. First, we examined the ultrastructure of the PES by electron microscopy (EM). In contrast to those untreated, cells exposed to PC(*O*-16:0/2:0) displayed large invaginations of the PM, which occasionally appeared as either a transversal cut of the PM invagination or potentially invaginations which have undergone scission and become cytoplasmic (Fig. 3A–F and Fig. S3A). The large invaginations of the PM present in PC(*O*-16:0/2:0) treated cells are reminiscent of the failed endocytic events that have previously been observed in *inp51Δ inp52Δ* cells [26]–[28]. The formation of these structures in the *inp51Δ inp52Δ* mutant is due to increased PtdIns(4,5)P_2_ levels as a result of reduced cellular PtdIns(4)P 5-phosphatase activity [28]. This phenomenon requires an intact actin cytoskeleton [28]. In contrast, pretreatment with Latrunculin A (Lat A), an actin depolymerizing agent, did not inhibit PES formation (Fig. 3G) and surprisingly we found that PC(*O*-16:0/2:0) treatment alone resulted in the disruption of the actin cytoskeleton (Fig. 3H). Similarly, deletion of *VRP1*, an actin associated protein required for cytoskeletal organization that suppresses the *inp51Δ inp52Δ* phenotype [29], did not affect PES formation or PC(*O*-16:0/2:0) toxicity (Fig. S3B and C). Combined these results strongly suggest that the PC(*O*-16:0/2:0)-dependent PES is distinct from the previously characterized PM invaginations seen in *inp51Δ inp52Δ* cells and that the PES formation occurs independently of the actin cytoskeleton. The actin-independency of PES formation could potentially be explained by an unregulated association of endocytic coat complex proteins or impaired exocytic vesicle fusion [30]. However, a RFP-fusion of Chc1, which associates at the PM independently of actin at sites of clathrin-mediated endocytosis [31], co-localized with GFP-2×PH^PLCδ^ at the PES in only 3% of cells (Fig. S3D). In addition, the localization of the exocyst component Exo70 was only modestly disrupted upon PC(*O*-16:0/2:0) treatment (Fig. S3E) and both Exo70-GFP or Sec3-GFP exhibited minimal co-localization with the PES marked by FM4-64 (Fig. S3F). These results indicate that the actin-independent events involved in PES formation likely do not involve the aberrant association of endocytic or exocytic proteins with the PM.

### PC(*O*-16:0/2:0) disrupts sphingolipid metabolism

**Figure 4:**
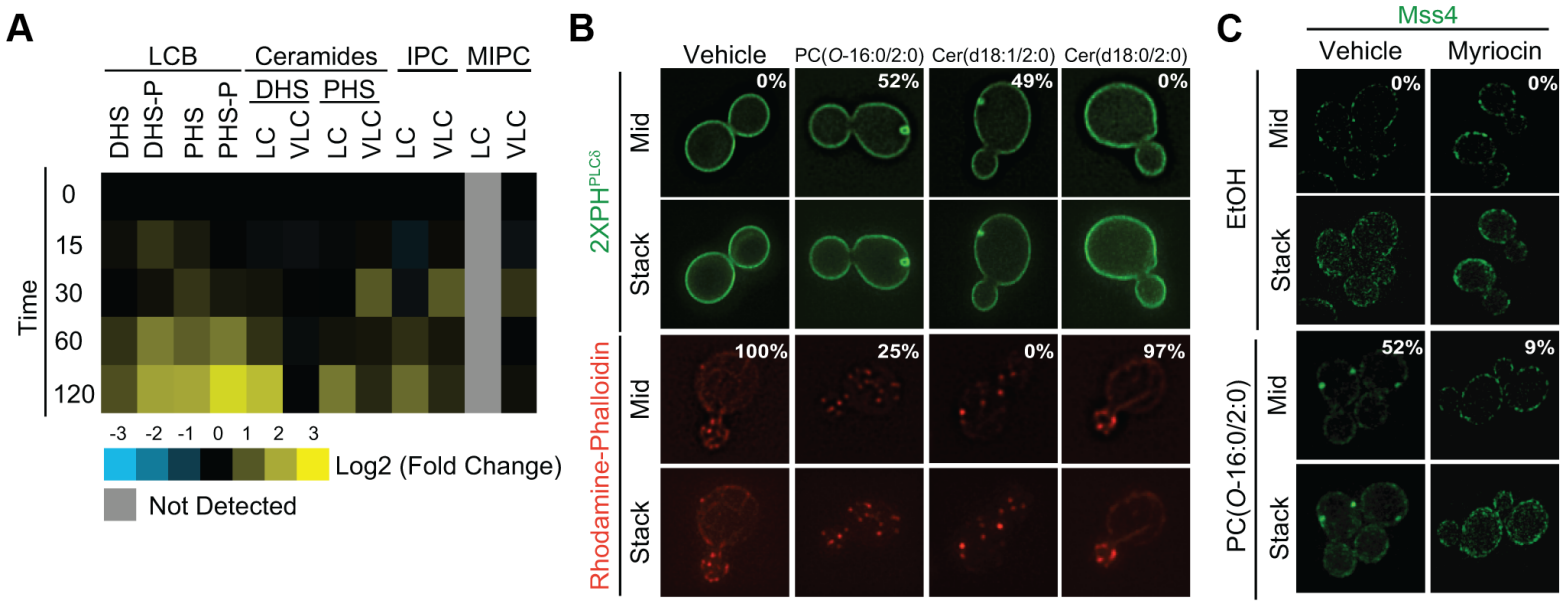
PC(*O*-16:0/2:0) disrupts sphingolipid metabolism leading to changes in Mss4-GFP localization. (**A**) **PC(** ***O*** **-16:0/2:0) treatment disrupts sphingolipid metabolism.** Wild type (BY4741) cells were treated with vehicle or PC(*O*-16:0/2:0) (20 µM) for the indicated times (min). Lipids were extracted and sphingolipid levels were quantified and expressed as a log_2_ fold change of PC(*O*-16:0/2:0) treated from vehicle treated control. LCB, long chain base; IPC, inositol phosphorylceramide; MIPC, mannosyl phosphorylceramide; DHS(-P), dihydrosphingosine (1-phosphate); PHS(-P), phytohydrosphingosine; LC, long chain (acyl chain is equal to or less than 22 carbons); VLC, very long chain (more than 22 carbons). (**B**) **Treatment with ceramide promotes PES formation and inhibits actin cytoskeleton polarization.** Wild type (BY4741) cells expressing GFP-2×PH^PLCδ^ were grown in YPD in the presence of vehicle (EtOH), PC(*O*-16:0/2:0), Cer(d18:1/2:0) or Cer(d18:0/2:0) (20 µM, 15 min) prior to imaging live or fixing and staining for acting cytoskeleton polarization as described in methods. The percentage of cells displaying a redistribution of the fluorescent reporter or proper actin polarization is reported in the inset of the respective figure. (**C**) **Inhibition of sphingolipid metabolism prevents the relocalization of Mss4.** Mss4-GFP (YKB2955) expressing cells were pretreated with vehicle or myriocin (5 µM) for 30 min and subsequently treated with vehicle or PC(*O*-16:0/2:0) (20 µM, 15 min) as previously done. Pretreatment with myriocin inhibited PC(*O*-16:0/2:0)-dependent changes in PES formation.

These findings suggested that Mss4 relocalization is a principal factor in PES formation and that perturbations to PM PtdIns(4,5)P_2_ distribution are critically involved in regulating the toxic effects of PC(*O*-16:0/2:0). How might PC(*O*-16:0/2:0) disrupt Mss4 localization? The association of this protein with the PM occurs through poorly defined processes and may involve a combination of protein-protein and lipid-protein interactions [30], [32], [33]. Interestingly, the only reported lipid factors mediating Mss4 localization to the PM are PtdIns(4)P and the complex sphingolipid mannose-inositol-phosphoceramide (MIPC) [30], [33]. Although the role of MIPC was not confirmed by a subsequent study [34], Gallego and co-workers have shown that Mss4 can bind to dihydrosphingosine-1 phosphate (DHS-1P) *in vitro* and that an extended treatment with an inhibitor of sphingolipid biosynthesis (myriocin, 2 h) results in relocalization of Mss4-GFP [32]. These results suggest that changes in sphingolipid levels can impact Mss4 localization. Therefore, we postulated that the biological consequences of PC(*O*-16:0/2:0) treatment may arise in response to the effects of PC(*O*-16:0/2:0) on either sphingolipid biosynthesis or catabolism. In agreement with this hypothesis, we observed a global accumulation of LCBs precursors, their phosphorylated derivatives (LCB-Ps), as well as immediate ceramide precursors and metabolites in cells treated with PC(*O*-16:0/2:0) for 90 min (Fig. 4A, Dataset 1 and Fig. S4B). Furthermore, a modest but significant increase in several unphosphorylated phytosphingosine (PHS) and dihydrosphingosine (DHS) species is evident at 15 min (Fig. S4B). We also report that these increases were not associated with a decrease in the abundance of complex sphingolipids suggesting that PC(*O*-16:0/2:0) does not induce their catabolism (Fig. 4A, Fig. S4D and Dataset S1). In addition, deletion of the *S. cerevisiae* enzyme required for catabolism of complex sphingolipids, *ISC1*, did not impact the effects of PC(*O*-16:0/2:0) upon cell growth, PES formation or sphingolipid levels indicating that PC(*O*-16:0/2:0) does not stimulate the breakdown of sphingolipids (Fig. S4B–D). Next, we sought to determine whether PC(*O*-16:0/2:0)-induced elevation in LCBs and/or ceramide levels contributed to PES formation. First, we directly assessed the effects of ceramide upon PES formation by treating cells with the cell permeable ceramide, Cer(d18:1/2:0), or a biologically inactive analog, Cer(d18:0/2:0) (Fig. 4B). Treatment with Cer(d18:1/2:0), but not Cer(d18:0/2:0) resulted in relocalization of PtdIns(4,5)P_2_ and depolarization of the actin cytoskeleton similar to what is observed upon exposure to PC(*O*-16:0/2:0) suggesting that elevated ceramide levels are sufficient to induce PES formation (Fig. 4B). To explore the role of PC(*O*-16:0/2:0)-induced accumulation of LCB and ceramide further, we next investigated the effects of myriocin, an inhibitor of sphingolipid biosynthesis [35] (Fig. S4A), on Mss4-GFP localization in PC(*O*-16:0/2:0) treated cells (Fig. 4C and S4A). To accomplish this, we first pretreated cells with myriocin for 30 minutes prior to exposing them to PC(*O*-16:0/2:0). Although longer exposure (2 h) to myriocin has been reported to impact Mss4-GFP localization [32], our short pretreatment with myriocin did not affect Mss4-GFP localization (Fig. 4C). Pretreatment with myriocin for this time period was sufficient to inhibit the relocalization of Mss4-GFP and PES formation induced by PC(*O*-16:0/2:0) (Fig. 4C and Fig. S4F). Combined, these results support the notion that PC(*O*-16:0/2:0) treatment promotes the accumulation of LCBs and ceramides, which in turn contribute to changes in the subcellular localization of Mss4-GFP, PtdIns(4,5)P_2_ and downstream signaling events including actin cytoskeleton polarization.

### PC(*O*-16:0/2:0) inhibits Tor2 signaling

**Figure 5:**
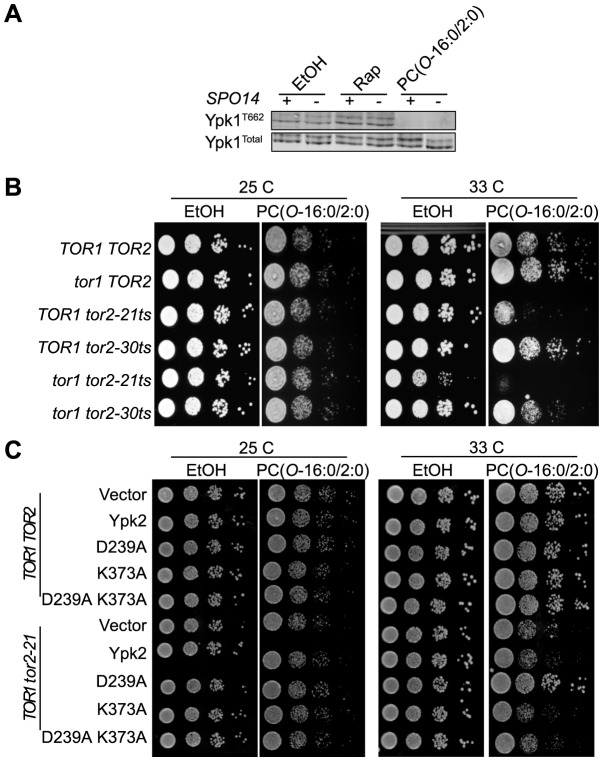
PC(*O*-16:0/2:0) inhibits TORC2 signaling. (**A**) **Phosphorylation of the TORC2 substrate Ypk1 is reduced following treatment.** TORC2-dependent Ypk1 (T662) phosphorylation status was assessed in whole cell extracts from vehicle (ethanol, EtOH), PC(*O*-16:0/2:0) (20 µM) or rapamycin (Rap, 200 ng/ml) treated wild type (YPH500) and *spo14*Δ (YKB2076) cells. Immunoblots were also probed with anti-sera for total Ypk1 to ensure equal loading. (**B**) ***tor2-21*** **mutants display increased sensitivity to PC(** ***O*** **-16:0/2:0).** Strains expressing plasmid borne wild type *TOR2* or the temperature sensitive (ts) alleles *tor2-21* or *tor2-30* in a *tor1*Δ, *tor2*Δ or a combined *tor1*Δ *tor2*Δ background were plated in 10-fold serial dilutions on YPD plates containing vehicle (EtOH) or PC(*O*-16:0/2:0) (3 µg/ml). Plates were incubated for 2 days at a permissive (25 C) or semi-permissive temperature (33 C). (**C**) **Overexpression of hyperactive Ypk2 suppresses sensitivity to PC(** ***O*** **-16:0/2:0).** Ypk2 wild type (Ypk2), hyperactive (D239A), kinase dead (K373A) and the double mutant (D239A and K373A) were transformed into wild type (SH100) and *tor2-21* (SH121) expressing cells. Growth was assessed following 2 days at permissive (25 C) and semi-permissive temperature (33 C) on plates containing vehicle (EtOH) or PC(*O*-16:0/2:0) (3 µg/ml).

We next sought to identify relevant signaling pathways which might be impacted by the effects of PC(*O*-16:0/2:0) upon sphingolipid metabolism and PM PtdIns(4,5)P_2_ localization. The target of rapamycin complex 2 (TORC2) was identified as a potential target because of its localization to the PM and the responsiveness of this signaling complex to changes in sphingolipid biosynthesis [36]–[38]. Furthermore, TORC2 has an established role in maintaining actin cytoskeleton polarization which is dependent upon the PM recruitment and phosphorylation of the homologous kinases Ypk1 and Ypk2 by the PtdIns(4,5)P_2_ binding proteins Slm1 and Slm2 [39], [40]. Utilizing a phospho-specific antibody recognizing a TORC2-dependent phosphorylation site on Ypk1 (T662) we determined that phosphorylation of endogenous Ypk1 was reduced in PC(*O*-16:0/2:0) suggesting that TORC2 signaling is inhibited by PC(*O*-16:0/2:0) (Fig. 5A) [37].

### A critical role for Tor2 and Ypk kinase signaling in PC(*O*-16:0/2:0) toxicity

Similar to mammalian cells, two distinct multiprotein complexes containing Tor activity, i.e. TORC1 and TORC2, are present in yeast. Unlike mammalian cells, however, yeast possess two *TOR* genes, *TOR1* and *TOR2*, with Tor1 nucleating the formation of TORC1 while Tor2 is able to nucleate both TORC1 and TORC2 [41]. Given that the phosphorylation of the TORC2 target Ypk1 is potently inhibited by PC(*O*-16:0/2:0), we next sought to determine whether Tor2 activity is required for preventing the growth inhibitory effects of PC(*O*-16:0/2:0). To assess the relative role of each Tor protein in buffering the growth inhibitory effects of PC(*O*-16:0/2:0), we made use of strains harboring the temperature sensitive *tor2-21* and *tor2-30* alleles alone or in combination with deletion of *TOR1* [42]. Whereas deletion of *TOR1* alone had no observable effect upon PC(*O*-16:0/2:0) sensitivity (Fig. 5B), the *tor2-21* strain exhibited a significant reduction in growth in the presence of PC(*O*-16:0/2:0) at a semi-permissive temperature. To further validate the role of TORC2 signaling in mediating PC(*O*-16:0/2:0) sensitivity we examined the effect of overexpressing the downstream target *YPK2* [40]. Consistent with a role for TORC2 in mediating the response to PC(*O*-16:0/2:0), we found that overexpression of a *YPK2* hyperactive allele (D239A), known to rescue lethality of TORC2 mutants [40], was able to restore growth of the *tor2-21* strain in the presence of PC(*O*-16:0/2:0). Comparatively, the wild type (Ypk2) and the kinase dead (K373A) variants [40] were unable to restore growth in the presence of reduced Tor2 function (Fig. 5C). Together, these results provide compelling evidence that TORC2 is inhibited in response to PC(*O*-16:0/2:0) treatment and that a reduction in TORC2 signaling is associated with an increased sensitivity to PC(*O*-16:0/2:0).

### Examining the mechanism underlying Tor2 inhibition by PC(*O*-16:0/2:0)

**Figure 6:**
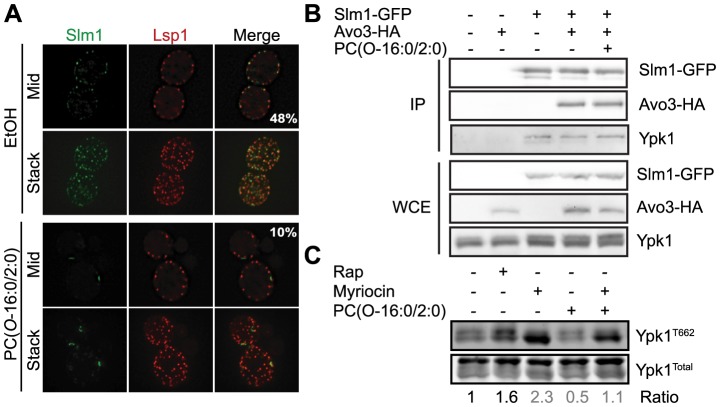
Relocalization of Slm1 by PC(*O*-16:0/2:0) does not mediate the inhibition of TORC2 signaling. (**A**) **PC(** ***O*** **-16:0/2:0) treatment relocalizes Slm1-GFP to foci.** The co localization of Slm1-GFP (YKB3035) with Lsp1-mcherry, an eisosome marker, was examined following treatment with either vehicle (EtOH) or PC(*O*-16:0/2:0) (20 µM) for 15 min. Numbers represent the percent of Slm1-GFP foci co-localizing with Lsp1-mcherry foci. (**B**) **PC(** ***O*** **-16:0/2:0) treatment does not affect TORC2 interactions.** The indicated strains were treated with either vehicle (EtOH) or PC(*O*-16:0/2:0) (20 µM) for 15 min. The interaction of Avo3-HA and endogenous Ypk1 with immunopurified (IP) Slm1-GFP was determined by immunoblotting with appropriate antibodies. Total levels of each protein were also examined in whole cell extracts (WCE). (**C**) **PC(** ***O*** **-16:0/2:0) still reduces Ypk1 phosphorylation in the presence of myriocin.** Wild type cells (TB50a) were pretreated with vehicle or myriocin (5 µM, 30 min) prior to adding rapamycin (Rap, 200 ng/ml) or PC(*O*-16:0/2:0) (20 µM). The ratio of TORC2-dependent Ypk1 phosphorylation to total Ypk1 was determined for each treatment condition and normalized to control. The mean is displayed below the representative blot (n = 2).

Since these results establish an important role for the TORC2-Ypk2 signaling in mediating the cellular response to PC(*O*-16:0/2:0), we investigated the potential mechanisms by which PC(*O*-16:0/2:0) might act to inhibit TORC2-dependent phosphorylation of Ypk1/2. The requirement for PtdIns(4,5)P_2_, PLD and Tor signaling in mediating PC(*O*-16:0/2:0) sensitivity presented the intriguing possibility that PLD-generated PA regulates Tor signaling in *S. cerevisiae* as previously reported for mTor [43]–[46]. However, deletion of *SPO14*, did not have noticeable effected the phosphorylation of endogenous Ypk1 suggesting that Spo14 does not impact TORC2 function in *S. cerevisiae* (Fig. 5A). Furthermore, knock out of *SPO14* exhibited a synthetic interaction with the *tor2-21* allele (Fig. S5). These results indicate that Spo14 and Tor2 likely act through parallel signaling pathways. Alternatively, the inhibition of Ypk1 phosphorylation in PC(*O*-16:0/2:0)-treated cells may be due to the direct inhibition of Tor kinase activity as was previously reported for cells with elevated glycerophosphocholine levels [47]. PC(*O*-16:0/2:0), however, did not inhibit the phosphorylation of recombinant GST-Ypk2 by immunopurified TORC2 suggesting PC(*O*-16:0/2:0) does not act as a direct inhibitor of Tor function *in vitro* and that a secondary mediator is required (Fig. S6A). Given that Ypk1/2 and TORC2 are normally localized to distinct subcellular compartments, however, the *in vitro* kinase assay likely does not fully recapitulate the constraints present *in vivo*. For example, phosphorylation of Ypk1/2 requires relocalization from the cytosol to the PM by TORC2 adaptor proteins Slm1/2 [48]. Interestingly, localization of Slm1/2 at the PM is itself partly dependent upon interactions with PtdIns(4,5)P_2_ [21], [34], [48]. We observed that PC(*O*-16:0/2:0) treatment disrupted the typical association of Slm1-GFP with eisosomes, a distinct spatially segregated compartment of the PM in *S. cerevisiae* [49], as indicated by the reduction in co-localization of Slm1-GFP with a tagged eisosome protein, Lsp1-mCherry (Fig. 6A). This redistribution of Slm1-GFP was not due to disruption of eisosome integrity but was associated with its appearance at the PES (Fig. S6B–D). Furthermore overexpression of Slm1 from a high copy plasmid enhanced growth compared to vector alone suggesting that Slm1-dependent signaling events are critically involved in mediating the cellular response to PC(*O*-16:0/2:0) (Fig. S6E). The correlation of Slm1 relocalization with increased LCBs and ceramides (Fig. 4 and S4) in PC(*O*-16:0/2:0)-treated cells is complementary with a previous report describing the impact of inhibiting sphingolpid metabolism upon the subcellular localization of Slm1 and Ypk1 phosphorylation [37]. Therefore, we next sought to investigate whether the relocalization of Slm1-GFP in PC(*O*-16:0/2:0) impaired the interaction of Ypk1 or TORC2. However, we found that the association of Slm1-GFP with HA-tagged TORC2 component Avo3 or untagged Ypk1 was not affected by PC(*O*-16:0/2:0) treatment suggesting the inhibition of TORC2 signaling does not require the redistribution of Slm1 to the PES (Fig. 6B). To support this conclusion we next investigated whether PES formation was necessary for the PC(*O*-16:0/2:0)-dependent inhibition of Ypk1 phosphorylation (Fig. 6C). Although pretreatment with myriocin alone increased Ypk1 phosphorylation ∼2.3 fold we observed that phosphorylation was similarly reduced (∼50%) in cells pretreated with either vehicle or myriocin upon treatment with PC(*O*-16:0/2:0) (Fig. 6C). Therefore, the inhibition of TORC2-dependent Ypk1 phosphorylation by PC(*O*-16:0/2:0) likely does not require the recruitment of Slm1 to the PES or a reduced interaction of Ypk1 with Slm1 or Avo3, indicating PC(*O*-16:0/2:0) inhibiting TORC2 through an previously undescribed mechanism.

## Discussion

**Figure 7:**
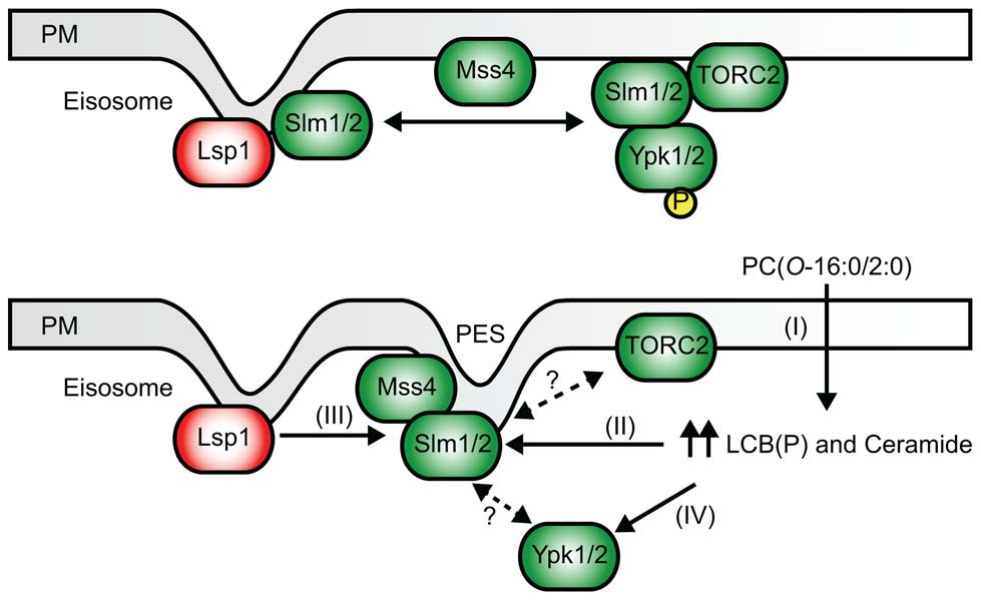
A simplified model of the impact of PC(*O*-16:0/2:0) on PtdIns(4,5)P_2_ and TOR signaling. Elevated PC(*O*-16:0/2:0) levels result in an increase in LCB(P) and ceramide species (I) which is associated with an altered localization of Mss4 and PtdIns (4,5)P_2_ (II) resulting in relocalization of Slm1, and presumably Slm2, from eisosomes to the PES (III) and a loss in TORC2-dependent Ypk1 phosphorylation without disrupting complex integrity (IV). Further work will be needed to determine if TORC2 components and/or Ypk1 are similarly recruited to the PES.

Aberrant glycerophosphocholine metabolism in AD leading to the intraneuronal accumulation of specific lipid second messengers, including PC(*O*-16:0/2:0) is linked to neuronal dysfunction, neurotoxicity, and accelerated cognitive decline [6], [50]–[52]. In this report we have used *S. cerevisiae* to further characterize the mechanisms underlying receptor-independent toxicity of PC(*O*-16:0/2:0). Our work suggests a model (Fig. 7) wherein exposure to toxic concentrations of PC(*O*-16:0/2:0) promotes the accumulation of LCBs and ceramides, which leads to changes in the subcellular localization of Mss4 and formation of PtdIns(4,5)P_2_ enriched invaginations of the PM. Ultimately the PC(*O*-16:0/2:0)-dependent remodeling of PtdIns(4,5)P_2_ affects downstream PtdIns(4,5)P_2_-dependent cellular processes such as PLD localization, which is critical for buffering against the toxic effects of PC(*O*-16:0/2:0) [9]. However, the inhibition of TORC2 by PC(*O*-16:0/2:0) also suggests that the toxic properties of PC(*O*-16:0/2:0) are only partly due to disruptions in PtdIns(4,5)P_2_ signaling and that this lipid impacts other signaling pathways through distinct second messengers that remain to be identified.

Given that PtdIns(4,5)P_2_ and downstream signaling events buffer against PC(*O*-16:0/2:0) toxicity, it was important to investigate the factors underlying the relocalization of Mss4-GFP and PES formation to elucidate potential endogenous mechanisms of neuroprotection. The molecular details that contribute to the localization of Mss4 into distinct phosphatidylinositol kinase or PIK patches in yeast are not completely understood. However, the availability of its substrate, PtdIns(4)P, a recently identified interacting partner Opy1 and sphingolipid biosynthesis have been implicated [30], [32], [33]. Our data suggests that the PC(*O*-16:0/2:0)-induced accumulation of LCBs and ceramides (precursor molecules in the sphingolipid biosynthetic pathway Fig. S4) are at least partly responsible for the changes in PM PtdIns(4,5)P_2_ distribution as treatment with myriocin, an inhibitor of sphingolipid biosynthesis, was sufficient to prevent the redistribution of Mss4-GFP and PES formation (Fig. 4 and Fig. S4). The mechanism by which the observed changes in LCBs and ceramide might regulate Mss4 PM localization are not clear but previous reports have suggested that both MIPC and dihydrosphingosine-1 phosphate (DHS-1P) can interact with Mss4 [32], [33]. The relocalization of Mss4-GFP, however, is likely not due to interactions with MIPC as neither the total levels of this lipid nor the abundance of individual species was significantly impacted by PC(*O*-16:0/2:0) at any time point (Fig. 4, Fig. S4 and Dataset 1). In contrast, the accumulation of one DHS and two PHS species displayed similar kinetics to the PtdIns(4,5)P_2_ redistribution and PES formation suggesting that these lipids may be involved in mediating the observed changes (Fig. S4B and Dataset 1). Certainly, this observation must be interpreted with caution as the reported *in vitro* interaction between Mss4 and LCBs has not been evaluated *in vivo* [32]. Furthermore, the role of LCB-Ps in mediating Mss4 localization at the PM must also be reconciled with the fact that LCB-Ps do not appear to be trafficked to the PM under normal circumstances [53]. Whether PC(*O*-16:0/2:0)-induced changes in Mss4-GFP localization are dependent upon the improper trafficking of LCB-Ps or another mechanism remains an open question in need of further study.

The spatial distribution of PtdIns(4,5)P_2_ at the PM is critical for regulating the activity of downstream signaling pathways. Our biochemical, genetic and cell biology based-assays suggest that the inhibition of Tor signaling plays a critical role in mediating the sensitivity to the toxic effects of PC(*O*-16:0/2:0) (Fig. 5 and 6). The results of our *in vitro* kinase assay do not identify PC(*O*-16:0/2:0) as a direct inhibitor of Tor kinase activity and suggests that another mechanism is responsible for the inhibition of Tor signaling (Fig. S6). How else might PC(*O*-16:0/2:0) inhibit Tor signaling? Although the cellular inputs which impinge upon Tor signaling are still being identified and the molecular mechanisms which translate these stimuli into activation/inhibition of Tor signaling are not completely understood, the TORC2-dependent phosphorylation of Ypk1/2 is sensitive to changes in PM PtdIns(4,5)P_2_ levels [48]. Our work demonstrating the relocalization of Slm1-GFP to the PES in response to PC(*O*-16:0/2:0) is consistent with previous reports describing the interactions of Slm proteins with PtdIns(4,5)P_2_ (Fig. 6 and S6) [1], [20]. Because our data indicate that relocalization of Slm1, and presumably Slm2, to the PES is not required for the inhibition of TORC2-dependent Ypk phosphorylation, they suggest that an additional mechanism(s) exists for the regulation of TORC2 signaling (Fig. 6).

Collectively, our results provide insight into how a disruption in phosphocholine metabolism signals network-wide lipid metabolic disturbances that may play defining roles into how neurons respond to accumulating Aβ_42_. Interestingly, accumulating evidence suggests that disruptions in both PtdIns(4,5)P_2_ signaling and ceramide metabolism are contributing factors in the neuronal cell dysfunction and death observed in AD [13]–[19]. Whether the disruptions in PtdIns(4,5)P_2_ signaling and ceramide metabolism homeostasis observed in neurons are dependent upon an increase in PC(*O*-16:0/2:0) concentrations is an intriguing question in need of further investigation.

## Materials and Methods

### Yeast strains, plasmids and media

The yeast strains and plasmids used in this study are listed in Table S2 and S3. Strains were generated by using a standard PCR-mediated gene insertion/deletion technique [54]. Cells were grown in standard YPD or SD medium supplemented with amino acids and all lipids were prepared by resuspending in either ethanol or methanol and storing under nitrogen gas.

### Cell growth and treatments

All strains were grown in YPD or minimal media supplemented with appropriate amino acids as required and treated with PC(*O*-16:0/2:0) (Enzo Life Sciences, BML-L100 or Avanti Polar Lipids, 878119P) at 20 µM for 15 minutes unless indicated otherwise. Media was supplemented with rapamycin (200 ng/ml) where indicated.

### Dot assays

Cells were grown in YPD or minimal media at 30 C to mid-log phase and resuspended to an OD_600_ of 0.1. Dot assays were performed by spotting 4 µL of ten-fold serial dilutions (OD_600_ = 0.1, 0.01, 0.001, 0.0001) onto YPD or minimal media selection plates containing the specified concentrations of ethanol, PC(*O*-16:0/2:0) or other chemical as indicated.

### Microscopy

For all microscopy experiments, overnight cultures grown at 30 C in YPD medium were re-suspended at a final OD_600_ of 0.2 and allowed to reach mid-log phase prior treatment and image acquisition. Live cell imaging was performed by briefly centrifuging the cells (800 g for 3 min), followed by resuspending in a minimal volume of growth media, spotting onto glass slides and coverslipping prior to imaging. All images were acquired using a Leica DMI 6000 florescent microscope (Leica Microsystems GmbH, Wetzler Germany), equipped with a Sutter DG4 light source (Sutter Instruments, California, USA), Ludl emission filter wheel with Chroma band pass emission filters (Ludl Electronic Products Ltd., NY, USA) and Hamamatsu Orca AG camera (Hamamatsu Photonics, Herrsching am Ammersee, Germany). Images were acquired at 0.2 µM steps using a 63× oil-immersion objective with a 1.4 numerical aperture. Deconvolution and analysis were performed using Velocity Software V4 (Perkin Elmer). For most images, representative images of the middle section and compressed image stack are shown. Numerical insets represent the indicated quantifications of at least 100 cells from 2 to 3 independent experiments unless indicated otherwise.

### Rhodamine-phalloidin staining

Early log phase cells were fixed by diluting 37% formaldehyde to a final concentration of 3.7% and incubating at 25 C for 10 minutes. Cells were subsequently pelleted (800 g for 3 min) and resuspended in PBS containing 3.7% formaldehyde and incubated for 1 hour. Cells were subsequently washed three times in PBS prior to staining with Rhodamine-conjugated phalloidin diluted in PBS containing 0.1% Tween on ice (20 Units/ml, Invitrogen) and cells were washed two times prior to imaging. For actin depolymerisation, Latrunculin A (5 µM, Tocris) was added as indicated prior to fixation and cell staining.

### Cell extract preparation

In all cases overnight cultures of yeast strains were diluted to an OD_600_ of 0.2 in YPD or appropriate minimal media and allowed to reach mid-log growth prior to harvesting. Cell pellets were resuspended in 200 µL of lysis buffer (20 mM HEPES, 150 mM NaCl, 2 mM EDTA with phosphatase and protease inhibitors and lysed by vortexing with glass beads. Ypk1 was examined in ethanol and PC(*O*-16:0/2:0) treated cells prepared as previously described [37]. Briefly, ice cold acetone was added to mid log phase cells and incubate on ice for 5 min. Cells were pelleted and washed two times in 5% acetone in PBS. Supernatant was removed and the cells pellets were dried under vacuum prior to lysis in urea buffer.

### Electron microscopy

Processing for electron microscopy was performed as previously described [55].

### TORC2 *in vitro* kinase assay

TORC2 was purified from RL127-1c cells. The cultures were grown to an OD_600_ of 5.0 in YPD (125 mL per assay point), chilled on ice for 30 minutes, collected, and washed. The cells were put into liquid nitrogen and ground up using a mortar and pestle. The powder was then resuspended in lysis buffer (1× Roche protease inhibitor +EDTA, 1 mM PMSF, phosphatase inhibitors, 5 mM CHAPS, 50 mM HEPES pH 7.5, 300 mM KCl), spun down, and 420 ul of prepared paramagnetic beads (Dynabeads M-270 Epoxy, coated with rabbit IgG; Sigma) were added to the cleared protein extracts. The tubes were subsequently rotated for 3 h at 4°C. Beads were collected by using a magnet and washed extensively with lysis buffer. The kinase reactions were performed in a final volume of 30 µl containing TORC2-coupled beads, 300 ng of Ypk2, 25 mM Hepes pH 7.0, 50 mM KCl, 4 mM MgCl2, 10 mM DTT, 0.5% Tween20, 1× Roche protease inhibitor-EDTA, 100 mM ATP, 5 mCi [γ-^32^P]-ATP and 1 µl of inhibitors at various concentrations. PAF was dissolved in EtOH and used at the indicated concentrations. Assays were started with addition of ATP, maintained at 30°C for 25 minutes and terminated by the addition of 7.5 µl of 5× SDS-PAGE buffer. Samples were heated at 65°C for 10 min; proteins were resolved in SDS-PAGE, stained with Sypro Ruby and analysed using a Bio-Rad Molecular Imager.

### Substrate preparation for *in vitro* kinase assays

GST-Ypk2 fusion proteins were expressed in *S. cerevisiae* from a pRS426 vector. Actively growing cells were induced for 3 hours with galactose (final concentration of 2%), chilled on ice for 30 minutes, and collected. The cells were put into liquid nitrogen and ground up using a mortar and pestle. The powder was then resuspended in lysis buffer (10% glycerol, 1×PBS, 0.5% Tween, 1× Roche protease inhibitor +EDTA, 1 mM PMSF, and phosphatase inhibitors) and the fusion protein was bound to and eluted from glutathione Sepharose 4B (GE Healthcare) following standard procedures. The supernatant was dialyzed against ( 50% glycerol, 1 mM DTT, 1 mM EDTA, 25 mM Tris pH 7.5, 50 mM NaCl), aliquotted, and frozen at −20°C.

### PC(*O*-16:0/2:0) treatment and lipid extraction

Cells at 0.6 OD_600_ were treated with 20 µM PAF or ethanol as a control. At T = 15 min, 30 min , 60 min and 120 min, 7.5 OD_600_ were harvested in glass tubes, washed with water and the pellet was extracted 3×1 ml in water∶Ethanol∶Diethyl Ether∶Pyridine∶NH4OH (15∶15∶5∶1∶0.018) at 65°C for 15 min each time. Avanti Polar Lipid MS standards (LM-6002) were added during the first extraction at 62.5 pmol/tube. The extracts were pooled and dried under N2, redissolved in 1 ml Chloroform with bath sonication, 1 ml Butanol was added and phospholipids were hydrolyzed for 30 min at 37°C after the addition of 200 µL 1 M KOH (in methanol). After hydrolysis, the extract was neutralized by the addition of 200 µL 1 M Acetic Acid (in Methanol). 1 ml Butanol saturated water was added, centrifuged to separate the phases and the upper aqueous layer was removed by aspiration, being careful not to disrupt the precipitate at the interface. This was repeated two more times after which the remaining lower phase was dried under N2. The dried lipid was redissolved in 0.5 ml LC/MS buffer A with bath sonication, spun to pellet insoluble material and the transferred to MS analysis vials.

### LC/MS analysis

The samples were analyzed on a Supelco Discovery Bio Wide Pore C18 (5 cm×2.1 mm, 5 uM) column at 40°C (50 mm) using an Agilent 1200 Series HPLC coupled to ABSciex QTRAP 4000 MS. The LCB(P)s were eluted using a binary solvent gradient of 0% B for 1 min, 25% at 4 min, 100% at 4.5 min and held at 100%B for 1.5 min, 0%B at 7 min. The LCB(P)s were detected in MRM mode,

### LC/MS buffers

MS buffer A: Tetrahydrofuran: Methanol: 10 mM Ammonium Formate (30∶20∶50) with 0.2% Formic Acid

MS buffer B: Tetrahydrofuran: Methanol: 10 mM Ammonium Formate (70∶20∶10) with 0.2% Formic Acid

## Supporting Information

Dataset S1 Results of lipid analysis by mass spectrometry.
(XLS)

Figure S1 Time course of PES formation. Redistribution of PtdIns(4,5)P_2_ by PC(*O*-16:0/2:0) is maximal by 15 min. Wild type (YPH500) cells expressing GFP-2×PH^PLCδ^ were treated with either vehicle (EtOH) or PC(*O*-16:0/2:0), 20 µM, 15 min, for the indicated times and the percentage of cells with redistributed GFP-2×PH^PLCδ^ quantified.
(EPS)

Figure S2 Characterization of PES formation. (A), (B) and (C) FM4-64 can be employed to visualize the PES. Wild type (BY4741) cells expressing GFP-2×PH^PLCδ^ were treated with either vehicle (EtOH) or PC(*O*-16:0/2:0) as done previously (20 µM, 15 min). Following treatment cells were collected into ice cold growth media and labeled with FM4-64 prior to imaging as described in methods. The percentage of cells displaying a redistribution of the fluorescent reporter GFP-2×PH^PLCδ^ is reported in the inset of the figure. Co-localization of the GFP-2×PH^PLCδ^ and FM4-64 signals upon treatment was quantified and is reported in the bar graph. (D) and (E) PES formation is reduced in *mss4-102* cells. The time course PES formation in wild type (SEY6210, circles) and *mss4-102* (AAY202, squares) strains grown at permissive (25 C, filled) and non-permissive temperatures (37 C, open) was investigated by treating cells with PC(*O*-16:0/2:0) (20 µM) for the indicated times followed by labeling with FM4-64 as described in methods. (F) *MSS4* overexpression improves growth in the presence of PC(*O*-16:0/2:0). Wild type (YKB1079) cells were transformed with the indicated 2μ plasmids (Vector, pRS426 or pRS426-Mss4-GFP) grown on YPD plates in the presence of vehicle (EtOH) or PC(*O*-16:0/2:0) at the indicated concentrations and growth assessed at 2 days. (G) Deletion of *INP51* improves growth in presence of PC(*O*-16:0/2:0). Wild type (BY4741), *inp51Δ* (YKB3412), *inp52Δ* (YKB343), *inp53Δ* (YKB3414) and *inp54Δ* (YKB3415) grown on YPD plates in the presence of vehicle (EtOH) or PC(*O*-16:0/2:0) at the indicated concentrations and growth assessed at 2 days. (H) PC(*O*-16:0/2:0)-induced PES formation occurs when cells are grown in galactose. Wild type (BY4741) cells were grown to in SD-Ura with galactose as the sole carbon source to replicate the growth conditions in Fig. 2D. Cells were treated with either vehicle (EtOH) or PC(*O*-16:0/2:0) as done previously (20 µM, 15 min). Following treatment cells were collected into ice cold growth media and labeled with FM4-64 prior to imaging as described in methods.
(EPS)

Figure S3 PES formation does not involve the actin bundling protein Vrp1, endocytic or exocytic components. (A) Quantification of PES formation in electron microscopy images. The percentage of cells with PES-like structures evident in wild type (BY4742) cells treated with PC(*O*-16:0/2:0) for 15 min assessed by electron microscopy. Numbers represent the mean of 100 cells from 3 separate grids. (B) *VRP1* is not required for PES formation. Wild type (BY4741) and *vrp1Δ* (YKB3017) cells expressing GFP-2×PH^PLCδ^ were grown in YPD in the presence of vehicle (EtOH) or PC(*O*-16:0/2:0) (20 µM, 15 min) prior to imaging live as previously done. The percentage of cells displaying a redistribution of the fluorescent reporter is reported in the inset of the figure. (C) Deletion of *VRP1* does not impact PC(*O*-16:0/2:0) toxicity. Wild type (YKB 1079), *vrp1Δ* (YKB3017) and *spo14Δ* (YKB3113) cells were spotted in 10-fold serial dilutions onto YPD plates in the presence of PC(*O*-16:0/2:0) (3 µg/ml) for 2 days at 30 C prior to imaging. (D) Clathrin heavy chain does not localize to the PES. A strain expressing Chc1-RFP (YKB2489) and GFP-2×PH^PLCδ^ were grown in YPD in the presence of vehicle (EtOH) or PC(*O*-16:0/2:0) (20 µM, 15 min) prior to imaging live as previously done. The percentage of cells displaying a redistribution of the fluorescent reporter is reported in the inset of the figure (GFP-2×PH^PLCδ^) as is the percentage of cells with co-localization of both markers (Merge). (E) Localization of Exo70-GFP is modestly impacted by PC(*O*-16:0/2:0). The distribution of GFP tagged Exo70 (YKB3417) was examined following treatment with vehicle (EtOH) or PC(*O*-16:0/2:0) (20 µM, 15 min). A modest increase in the localization of Exo70-GFP to sites other than an incipient bud site, bud neck or bud tip was observed in treated cells. (F) Exocyst components co-localize with the PES in a minority of cells. Co-localization of GFP tagged Sec3 (YKB3416) and Exo70 (YKB3417) with FM4-64 was assessed following treatment with PC(*O*-16:0/2:0) (20 µM, 15 min) by scoring the percentage of cells where the GFP and FM4-64 signals co-localized in cells where a PES was evident.
(EPS)

Figure S4 *ISC1* is not required for PC(*O*-16:0/2:0)-dependent disruptions in sphingolipid metabolism and PES formation. (A) A simplified schematic of the sphingolipid biosynthetic pathway in yeast. (B) Several LCB species are significantly elevated at 15 min. Analysis of the individual phosphorylated and unphosphorylated long chain base species represented at 15 min of treatment PC(*O*-16:0/2:0) (20 µM, closed bars) reveals a significant increase in comparison to vehicle (EtOH, open bars) (* indicates p<0.05, multiple t-tests, error bars represent SEM, n = 3). Data are also represented in Fig. 4A and Dataset 1. (C) Deletion of *ISC1 does not impact PC(O-16:0/2:0) toxicity.* Wild type (YKB1079), *isc1Δ* (YKB3265) cells were spotted in 10-fold serial dilutions onto YPD plates in the presence of PC(*O*-16:0/2:0) (6 µg/ml) for 2 days at 30 C prior to imaging. (D) *ISC1* is not required for PES formation. An *isc1Δ* (YKB3265) strain expressing GFP-2×PH^PLCδ^ was grown in YPD in the presence of vehicle (EtOH) or PC(*O*-16:0/2:0) (20 µM, 15 min) prior to imaging live as previously done. The percentage of cells displaying a redistribution of the fluorescent reporter is reported in the inset of the figure. (E) Catabolism of complex sphingolipids is not evident in PC(*O*-16:0/2:0) treated cells. Wild type (BY4741) and *isc1Δ* (YKB3265) cells were treated with vehicle or PC(*O*-16:0/2:0) (20 µM) for 90 min. Lipids were extracted as described in methods and sphingolipid levels were quantified and expressed as a log_2_ fold change of PC(*O*-16:0/2:0) treated from vehicle treated control. IPC, inositol phosphorylceramide; MIPC, mannosyl phosphorylceramide; MIP2C, mannosyl diinositol phosphorylceramide. (n = 1). (F) Inhibition of sphingolipid metabolism with myriocin prevents PES formation. Wild type (BY4741) expressing cells were pretreated with vehicle or myriocin (5 µM) for 30 min and subsequently treated with vehicle or PC(*O*-16:0/2:0) (20 µM, 15 min) as previously done. Following treatment cells were collected into ice cold growth media and labeled with FM4-64 prior to imaging as described in methods. The percentage of cells displaying a redistribution of the fluorescent reported is indicated in the inset of the figure.
(EPS)

Figure S5 Deletion of *SPO14* and/or *TOR2* confer sensitivity to PC(*O*-16:0/2:0) through distinct mechanisms. *SPO14* and *TOR2* exhibit a synthetic genetic interaction. Synthetic interactions between *SPO14* and *TOR1 and TOR2* were examined. The indicated strains were grown, diluted and spotted onto YPD plates as described above. Growth was assessed after 2 days at the permissive (25 C) and semi permissive (33 C) temperatures.
(EPS)

Figure S6 Investigating mechanisms of PC(*O*-16:0/2:0)-dependent TORC2 inhibition. (A) PC(*O*-16:0/2:0) does not inhibit TORC2 kinase activity. *In vitro* kinase assay of immunoprecipitated TOR2 was performed using a yeast purified kinase dead GST-Ypk2 as substrate, where either DMSO, Wortmannin (WM), ethanol (EtOH), or differing concentrations of PC(*O*-16:0/2:0) (20, 10, 1 uM) were added to the kinase reaction. (B) Eisosome integrity is not impacted by PC(*O*-16:0/2:0). Localization of Pil1-GFP (YKB3112), a protein required for eisosome integrity, was similarly examined in vehicle (EtOH) and PC(*O*-16:0/2:0) (20 µM) treated cells (15 min) to examine the impact upon eisosomes. (C) and (D) Slm1-GFP localizes to the PES in PC(*O*-16:0/2:0) treated cells. A Slm1-2×RFPmars (TWY2560) strain expressing GFP-2×PH^PLCδ^ was treated with either vehicle (EtOH) or PC(*O*-16:0/2:0) (20 µM, 15 min) and localization of the two probes was assessed. Representative images of the mid section (Mid) and the compressed stack (Stack) are shown. Enrichment of GFP-2×PH^PLCδ^ and Slm1-2×RFPmars at the PES sites in individual cells was calculated using imageJ [56] as follows: average PES associated pixel intensity per unit area/total plasma membrane associated pixel intensity per unit area. (n = 25 cells) (E) Overexpression of Slm1 improves growth in the presence of PC(*O*-16:0/2:0). Wild type cells (BY4741) containing an empty vector or expressing Slm1 from a high copy plasmid were spotted in 10-fold serial dilutions onto SC-Leu plates in the presence of vehicle (EtOH) or PC(*O*-16:0/2:0) at the indicated concentrations for 2 days at 30 C prior to imaging.
(EPS)

Table S1 List of cellular stresses examined for effects on GFP-2×PH^PLCδ^ localization.
(DOC)

Table S2 List of yeast strains used.
(DOC)

Table S3 List of plasmids used.
(DOC)
