# Supplementary material for: A Neurotoxic Glycerophosphocholine Impacts PtdIns-4, 5-Bisphosphate and TORC2 Signaling by Altering Ceramide Biosynthesis in Yeast
Source: PLoS Genet. 2014 Jan 23;10(1):e1004010. doi: 10.1371/journal.pgen.1004010 (PMC3900389; doi:10.1371/journal.pgen.1004010)
Supplement: Table S1 — List of cellular stresses examined for effects on GFP-2×PHPLCδ localization. (DOC) [file pgen.1004010.s008.doc]

**Table S1. List of cellular stresses examined for effects on GFP-2XPH^PLCδ^ localization.**

| Substance | Known Cellular Effect | Concentration | Growth Inhibition^1^ | Source |
| --- | --- | --- | --- | --- |
| Rapamycin | Tor Inhibition | 200 ng/ml | ND | Sigma (R0395) |
| Wortmannin | PI3K Inhibitor | 5 µM | ND | LC Laboratories (W-2990) |
| Cycloheximide | Inhibition of Protein Synthesis | 25 mg/ml | ND | Sigma (C7698) |
| Nocodazole | Microtubule Disruption | 10 mg/ml | ND | Sigma (M1404) |
| DTT | ER Stress | 1 mM | ND | Sigma(D9779) |
| NaCl | Hyperosmotic Stress | 1 M | ND | Wisent(600-082) |
| 37 C | Heat Stress | - | ND | - |
| 50 C | Heat Stress | - | ND | - |
| PC(*O*-16:0/0:0) | Unknown | 20 µM | Yes | Avanti (878110P) |
| PC(*O*-16:0/2:0) | PtdIns(4,5)P_2_ Relocalization | 20 µM | Yes | Avanti (878119P) |
| PC(16:0/0:0) | Unknown | 20 µM | Yes | Avanti (855675P) |
| PC(16:0/2:0) | Unknown | 20 µM | ND | Avanti (880622C) |
| PC(16:0/16:0) | Unknown | 20 µM | ND | Avanti (850355P) |
| PC(*O*-18:0/2:0) | Unknown | 20 µM | Yes | Avanti (878114P) |
| PC(*O*-18:0/0:0) | Unknown | 20 µM | Yes | Avanti (878120P) |
| PC(18:0/0:0) | Unknown | 20 µM | Yes | Avanti (855775P) |
| PC(*O*-18:1(9Z)e/0:0) | Unknown | 20 µM | Yes | Avanti (878126P) |
| PC(18:1(9Z)/0:0) | Unknown | 20 µM | Yes | Avanti (845875P) |
| PC(P-18:1/0:0) | Unknown | 20 µM | Yes (>80 μM) | Avanti (852465P) |
| PC(18:0/ 20:4(5Z,8Z,11Z,14Z)) | Unknown | 20 µM | ND | Avanti (850469C) |
| Cer(d18:0/2:0) | Unknown | 20 µM | ND | Sigma (A7191) |
| Cer(d18:0/0:0) | Unknown | 20 µM | ND | Sigma (C7980) |

The table includes descriptions of the substances, the cellular processes targeted (if known), maximal concentrations tested for effects on localization of the GFP-2XPH^PLCδ^ probe and the impact on growth if determined. (1) Growth inhibition was assessed in triplicate cultures by measuring the OD_600_ every 0.5 hours for 19.5 hours for cells grown at 30 C in the presence of each lipid from a starting OD_600_ of 0.05 (n=2 or 3). Inhibition was deemed evident if maximal OD_600_ levels were reduced compared to vehicle treated cells (n= 2 or 3). ND – not determined.
