# Supplementary material for: A Neurotoxic Glycerophosphocholine Impacts PtdIns-4, 5-Bisphosphate and TORC2 Signaling by Altering Ceramide Biosynthesis in Yeast
Source: PLoS Genet. 2014 Jan 23;10(1):e1004010. doi: 10.1371/journal.pgen.1004010 (PMC3900389; doi:10.1371/journal.pgen.1004010)
Supplement: Table S2 — List of yeast strains used. (DOC) [file pgen.1004010.s009.doc]

**Supplementary Table S2. List of yeast strains used.**

| Name | Genotype | Source |
| --- | --- | --- |
| YPH500 | ***MAT****α ade2-101 his3-Δ200 leu2-Δ1 lys2-801 trp1-Δ63 ura3-52* |  |
| YKB2076 | ***MATα*** *ade2-101 his3-Δ200 leu2-Δ1 lys2-801 trp1-Δ63 ura3-52 spo14Δ::TRP* |  |
| SH100 | ***MATa*** *leu2-3,112 ura3-52 rme1 trp1 his4 GAL+ ade2 tor2::ADE2-3/Ycplac111::TOR2* |  |
| SH121 | ***MATa*** *leu2-3,112 ura3-52 rme1 trp1 his4 GAL+ ade2 tor2::ADE2-3/Ycplac111::tor2-21* |  |
| SH130 | ***MATa*** *leu2-3,112 ura3-52 rme1 trp1 his4 GAL+ ade2 tor2::ADE2-3/Ycplac111::tor2-30* |  |
| SH200 | ***MATa*** *leu2-3,112 ura3-52 rme1 trp1 his3 GAL+ ade2 tor1::HIS3-3 tor2::ADE2-3/Ycplac111::TOR2* |  |
| SH221 | ***MATa*** *leu2-3,112 ura3-52 rme1 trp1 his3 GAL+ ade2 tor1::HIS3-3 tor2::ADE2-3/Ycplac111::tor2-21* |  |
| SH230 | ***MATa*** *leu2-3,112 ura3-52 rme1 trp1 his3 GAL+ ade2 tor1::HIS3-3 tor2::ADE2-3/Ycplac111::tor2-21* |  |
| YKB2829 | ***MATa*** *leu2-3,112 ura3-52 rme1 trp1 his4 GAL+ ade2 tor2::ADE2-3/Ycplac111::TOR2 spo14Δ::NATMX* | This study |
| YKB2831 | ***MATa*** *leu2-3,112 ura3-52 rme1 trp1 his4 GAL+ ade2 tor2::ADE2-3/Ycplac111::to2-21 spo14Δ::NATMX* | This study |
| YKB2830 | ***MATa*** *leu2-3,112 ura3-52 rme1 trp1 his3 GAL+ ade2 tor1::HIS3-3 tor2::ADE2-3/Ycplac111::TOR2 spo14Δ::NATMX* | This study |
| YKB2832 | ***MATa*** *leu2-3,112 ura3-52 rme1 trp1 his3 GAL+ ade2 tor1::HIS3-3 tor2::ADE2-3/Ycplac111::tor2-21 spo14Δ::NATMX* | This study |
| YKB3035 | ***MATa*** *ade2-101 LSP1-mCherry::HIS3 SLM1-GFP::HIS3* | This study |
| YKB3112 | ***MATa*** *his3Δ1 leu2Δ0 met15Δ0 ura3Δ0 PIL1-GFP::HIS3* | This study |
| YKB2955 | ***MATa*** *his3Δ1 leu2Δ0 met15Δ0 ura3Δ0 MSS4-GFP::HIS3* | This study |
| SEY6210 | ***MATα*** *leu2-3, 112,ura3-52 his3-Δ200 trp1-Δ901 lys2-801 suc2-Δ9* |  |
| AAY202 | ***MATα*** *leu2-3, 112,ura3-52 his3-Δ200 trp1-Δ901 lys2-801 suc2-Δ9*  *mss4Δ::HIS3/Ycpla:: mss4-102* |  |
| AAY102 | ***MATα*** *leu2-3, 112,ura3-52 his3-Δ200 trp1-Δ901 lys2-801 suc2-Δ9 stt4Δ::HIS3/pRS415stt4-4* |  |
| BY4741 | ***MATa*** *his3Δ1 leu2Δ0 lys2Δ0 met15Δ0 ura3Δ0* |  |
| YKB3412 | ***MATa*** *his3Δ1 leu2Δ0 lys2Δ0 met15Δ0 ura3Δ0 inp51Δ::KANMX* | This Study |
| YKB3413 | ***MATa*** *his3Δ1 leu2Δ0 lys2Δ0 met15Δ0 ura3Δ0 inp52Δ::KANMX* | This Study |
| YKB3414 | ***MATa*** *his3Δ1 leu2Δ0 lys2Δ0 met15Δ0 ura3Δ0 inp53Δ::KANMX* | This Study |
| YKB3415 | ***MATa*** *his3Δ1 leu2Δ0 lys2Δ0 met15Δ0 ura3Δ0 inp54Δ::KANMX* | This Study |
| YKB3017 | ***MATa*** *his3Δ1 leu2Δ0 lys2Δ0 met15Δ0 ura3Δ0 vrp1Δ::KANMX* | This study |
| YKB3113 | ***MATa*** *his3Δ1 leu2Δ0 lys2Δ0 met15Δ0 ura3Δ0 spo14Δ::KANMX* | This study |
| YKB2489 | ***MATα*** *his3Δ1 leu2Δ0 lys2Δ0 ura3Δ0*  *CHC1-RFP::KANMX6* |  |
| YKB3416 | ***MATa*** *his3Δ1 leu2Δ0 met15Δ0 ura3Δ0 SEC3-GFP::HIS3* |  |
| YKB3417 | ***MATa*** *his3Δ1 leu2Δ0 met15Δ0 ura3Δ0 EXO70-GFP::HIS3* |  |
| YKB3265 | ***MATa*** *his3Δ1 leu2Δ0 lys2Δ0 met15Δ0 ura3Δ0*  *isc1Δ::KANMX* | This study |
| TB50a | ***MATa*** *leu2 ura3 rme1 trp1 his3delete GAL+ HMLa* |  |
| TWY2560 | ***MATa*** *ura3 trp1 leu2 his3 ade2 can1-100* *SLM1-2xRFPmars::Nat* |  |
| RL127-1c | ***MATa*** *leu2 ura3 rme1 trp1 his3delete GAL+ HMLa AVO3-TAP::TRP1* | This study |
| RL170-2c | ***MATa*** *leu2 ura3 rme1 trp1 his3delete GAL+ HMLa TCO89-TAP::TRP1* | This study |
| YKB2956 | ***MATa*** *his3Δ1 leu2Δ0 met15Δ0 ura3Δ0*  *SLM1-GFP::HIS3* | This study |
| YKB3418 | ***MATa*** *his3Δ1 leu2Δ0 met15Δ0 ura3Δ0*  *AVO3-HA::KANMX* | This study |
| YKB3419 | ***MATa*** *his3Δ1 leu2Δ0 met15Δ0 ura3Δ0*  *SLM1-GFP::HIS3 AVO3-HA::KANMX* | This study |

1. Sikorski RS & Hieter P (1989) A system of shuttle vectors and yeast host strains designed for efficient manipulation of DNA in Saccharomyces cerevisiae. *Genetics* 122(1):19-27.

2. Kennedy MA*, et al.* (2011) Srf1 is a novel regulator of phospholipase D activity and is essential to buffer the toxic effects of C16:0 platelet activating factor. *PLoS genetics* 7(2):e1001299.

3. Helliwell SB, Howald I, Barbet N, & Hall MN (1998) TOR2 is part of two related signaling pathways coordinating cell growth in Saccharomyces cerevisiae. *Genetics* 148(1):99-112.

4. Robinson JS, Klionsky DJ, Banta LM, & Emr SD (1988) Protein sorting in Saccharomyces cerevisiae: isolation of mutants defective in the delivery and processing of multiple vacuolar hydrolases. *Molecular and cellular biology* 8(11):4936-4948.

5. Stefan CJ, Audhya A, & Emr SD (2002) The yeast synaptojanin-like proteins control the cellular distribution of phosphatidylinositol (4,5)-bisphosphate. *Molecular biology of the cell* 13(2):542-557.

6. Audhya A, Foti M, & Emr SD (2000) Distinct roles for the yeast phosphatidylinositol 4-kinases, Stt4p and Pik1p, in secretion, cell growth, and organelle membrane dynamics. *Molecular biology of the cell* 11(8):2673-2689.

7. Brachmann CB*, et al.* (1998) Designer deletion strains derived from Saccharomyces cerevisiae S288C: a useful set of strains and plasmids for PCR-mediated gene disruption and other applications. *Yeast* 14(2):115-132.

8. Huh WK*, et al.* (2003) Global analysis of protein localization in budding yeast. *Nature* 425(6959):686-691.

9. Beck T & Hall MN (1999) The TOR signalling pathway controls nuclear localization of nutrient-regulated transcription factors. *Nature* 402(6762):689-692.

10. Berchtold D*, et al.* (2012) Plasma membrane stress induces relocalization of Slm proteins and activation of TORC2 to promote sphingolipid synthesis. *Nature cell biology* 14(5):542-547.
