# Supplementary material for: A Neurotoxic Glycerophosphocholine Impacts PtdIns-4, 5-Bisphosphate and TORC2 Signaling by Altering Ceramide Biosynthesis in Yeast
Source: PLoS Genet. 2014 Jan 23;10(1):e1004010. doi: 10.1371/journal.pgen.1004010 (PMC3900389; doi:10.1371/journal.pgen.1004010)
Supplement: Table S3 — List of plasmids used. (DOC) [file pgen.1004010.s010.doc]

**Table S3**. List of plasmids used.

| Plasmid | Source |
| --- | --- |
| YEp352 YPK2-HA |  |
| YEp352 YPK2^D239A^-HA |  |
| YEp352 YPK2^K373A^-HA |  |
| YEp352 YPK2^D239A K373A^-HA |  |
| pRS416 |  |
| pEGH pGAL1-10::GST-6xHIS-INP51 |  |
| pEGH pGAL1-10::GST-6xHIS-INP52 |  |
| pEGH pGAL1-10::GST-6xHIS-INP54 |  |
| pRS426 | (2) |
| pRS426 Mss4-GFP |  |
| pRS425 | (2) |
| pRS425 Slm1 |  |
| pRS426 GST-YPK2^KD^ | This Study |

1. Kamada Y*, et al.* (2005) Tor2 directly phosphorylates the AGC kinase Ypk2 to regulate actin polarization. *Molecular and cellular biology* 25(16):7239-7248.

2. Sikorski RS & Hieter P (1989) A system of shuttle vectors and yeast host strains designed for efficient manipulation of DNA in Saccharomyces cerevisiae. *Genetics* 122(1):19-27.

3. Zhu H*, et al.* (2001) Global analysis of protein activities using proteome chips. *Science* 293(5537):2101-2105.

4. Audhya A & Emr SD (2003) Regulation of PI4,5P2 synthesis by nuclear-cytoplasmic shuttling of the Mss4 lipid kinase. *The EMBO journal* 22(16):4223-4236.

5. Magtanong L*, et al.* (2011) Dosage suppression genetic interaction networks enhance functional wiring diagrams of the cell. *Nature biotechnology* 29(6):505-511.
